# Classification prediction of pancreatic cystic neoplasms based on radiomics deep learning models

**DOI:** 10.1186/s12885-022-10273-4

**Published:** 2022-11-29

**Authors:** Wenjie Liang, Wuwei Tian, Yifan Wang, Pan Wang, Yubizhuo Wang, Hongbin Zhang, Shijian Ruan, Jiayuan Shao, Xiuming Zhang, Danjiang Huang, Yong Ding, Xueli Bai

**Affiliations:** 1 Department of Radiology, The First Affiliated Hospital, Zhejiang University School of Medicine, Zhejiang, Hangzhou China; 2 College of Information Science & Electronic Engineering, School of Micro-Nano Electronics, Zhejiang University, Zheda Road, Zhejiang, Hangzhou China; 3 Department of Radiology, Yiwu Central Hospital, Yiwu, Zhejiang, China; 4 College of Information Science & Electronic Engineering, Zhejiang University, Zhejiang, Hangzhou China; 5 Polytechnic Institute, Zhejiang University, Zhejiang, Hangzhou China; 6 Department of Pathology, The First Affiliated Hospital, Zhejiang University School of Medicine, Zhejiang, Hangzhou China; 7 Department of Radiology, Taizhou First People’s Hospital, Taizhou, Zhejiang, China; 8 Department of Hepatobiliary and Pancreatic Surgery, The First Affiliated Hospital, Zhejiang University School of Medicine, Qingchun Road, Zhejiang, Hangzhou China; 9 Zhejiang Provincial Key Laboratory of Pancreatic Disease, The First Affiliated Hospital, Zhejiang University School of Medicine, Zhejiang, Hangzhou China

**Keywords:** Radiomics, Computed tomography, Pancreatic cystic neoplasm, Deep learning

## Abstract

**Background:**

Preoperative prediction of pancreatic cystic neoplasm (PCN) differentiation has significant value for the implementation of personalized diagnosis and treatment plans. This study aimed to build radiomics deep learning (DL) models using computed tomography (CT) data for the preoperative differential diagnosis of common cystic tumors of the pancreas.

**Methods:**

Clinical and CT data of 193 patients with PCN were collected for this study. Among these patients, 99 were pathologically diagnosed with pancreatic serous cystadenoma (SCA), 55 were diagnosed with mucinous cystadenoma (MCA) and 39 were diagnosed with intraductal papillary mucinous neoplasm (IPMN). The regions of interest (ROIs) were obtained based on manual image segmentation of CT slices. The radiomics and radiomics-DL models were constructed using support vector machines (SVMs). Moreover, based on the fusion of clinical and radiological features, the best combined feature set was obtained according to the Akaike information criterion (AIC) analysis. Then the fused model was constructed using logistic regression.

**Results:**

For the SCA differential diagnosis, the fused model performed the best and obtained an average area under the curve (AUC) of 0.916. It had a best feature set including position, polycystic features (≥6), cystic wall calcification, pancreatic duct dilatation and radiomics-DL score. For the MCA and IPMN differential diagnosis, the fused model with AUC of 0.973 had a best feature set including age, communication with the pancreatic duct and radiomics score.

**Conclusions:**

The radiomics, radiomics-DL and fused models based on CT images have a favorable differential diagnostic performance for SCA, MCA and IPMN. These findings may be beneficial for the exploration of individualized management strategies.

**Supplementary Information:**

The online version contains supplementary material available at 10.1186/s12885-022-10273-4.

## Background

Pancreatic cystic neoplasms (PCNs) are a set of heterogeneous pancreatic cystic tumors with various biological behaviors [1]. The detection rate of PCN in the general population depends on the imaging method [2]. The detection rate of magnetic resonance imaging (MRI) is even close to 50% [3].

Common PCNs include serous cystic neoplasms (SCNs) and non-SCNs (mucinous cystic neoplasms, MCNs; intraductal papillary mucinous neoplasms, IPMNs; etc.) [1–3]. Among them, SCA, MCA and IPMN make up the majority of cases of PCNs. SCA has no tendency to undergo malignant transformation. Hence, follow-up observation is usually recommended in the clinical guidelines [2, 4]. Unlike SCA, MCN and IPMN have certain malignant transformation rates. Surgical resection is required for the latter to achieve a favorable prognosis [2, 4–6]. Therefore, there is an urgent demand to obtain an accurate preoperative diagnosis of PCNs for the formulation of individualized treatment schemes and follow-up strategies.

Currently, CT and MRI are the primary imaging methods for the evaluation of PCNs, depending on the morphological features of the tumors [2, 7]. Although PCNs with typical characteristics can be identified, those with atypical PCNs bring challenges to individualized clinical diagnosis and treatment. The requirements for individualized evaluation of patients with PCNs cannot be fully satisfied by morphological features.

In recent years, novel radiomics and radiogenomics studies have provided new ideas for the evaluation of tumors through deep learning and machine learning (ML) algorithms [8–13]. Recent studies have found that radiomics models contribute greatly to the individualized evaluation of pancreatic lesions, such as tumor detection, classification, differentiation, and antitumor drug effect prediction [14–18]. In addition, pancreatic cystic lesions have been categorized using radiomics methods in multiple studies [19, 20]. Although these findings confirmed the feasibility of radiomics for the assessment of pancreatic cystic lesions [21–28], the robustness of these radiomics diagnostic models may be limited due to the relatively small datasets included in most studies. Therefore, the accumulation of additional research data is required for the study of pancreatic cystic lesions.

This study aimed to construct diagnostic models based on radiomics and deep learning algorithms to differentiate between SCA and non-SCA, and between MCA and IPMN for the individualized evaluation of three common cystic neoplasms.

## Methods

### Ethical information and data collection

This retrospective study was approved by the ethics committee of our institution (the First Affiliated Hospital of Zhejiang University). The inclusion criteria were: (1) The patient underwent surgical resection and was pathologically diagnosed with SCA, MCA or IPMN. (2) Unenhanced and contrast-enhanced CT scan data for the pancreas taken within a month before surgery were available. The exclusion criteria were: (1) The region of interest (ROI) could not be determined due to poor image quality. (2) The CT or pathology data were incomplete. Data from 193 patients with PCNs who were treated at this hospital from January 2012 to January 2020 were collected for this study, including 99 patients diagnosed with SCA, 55 diagnosed with MCA, and 39 diagnosed with IPMN. The clinical data of these patients were all collected by one surgeon. The flowchart of data collection process in our study is shown in Fig. S1.

### Radiological feature analysis

In this study, the morphological features of the pancreatic cystic lesions were evaluated. The radiological features included the tumor position, maximum diameter, unilocular/ multilocular cysts, lobulation, polycystic features (≥2, ≥6), nodular soft tissue, cystic wall calcification, communication with the pancreatic duct, pancreatic duct dilatation, and peripancreatic lymph node enlargement. These features were evaluated by two radiologists (with 8 years and 10 years abdominal diagnosis experience in CT images, respectively) who were unaware of the pathology results. If there were inconsistencies in the radiological evaluation, a senior radiologist participated in the evaluation. Thus, the morphological features of the pancreatic cystic lesions were obtained from the CT images.

### Radiomics analysis

#### Image segmentation

This radiomics study was conducted based on arterial phase images from pancreas CT data. ITK-SNAP (v3.6.0) software was used to manually segment the ROI. Two radiologists performed segmentation on the cross-sectional layer with the largest area of the tumor, and a senior radiologist participated in the segmentation review.

#### Radiomics feature extraction

In an attempt to normalize the radiomics features, isotropic resampling and uniform quantization of image gray levels were performed. The radiomics features extracted in this study included three categories: global histogram features, second-order texture features and high-order filtering features. High-order filtering features include wavelet features and local binary pattern (LBP) features. LBP image features were obtained by LBP decomposition and reconstruction through rotation invariance.

#### Radiomics feature selection and model construction

To reduce the error of ROI segmentation, the ROI was segmented twice by different radiologists. Images segmented by the two radiologists were collected and different sets of features were extracted through ROIs. The intraclass correlation coefficient (ICC) was used to measure the repeatability of the different sets of radiomics features. Radiomics features with ICC values over the threshold were regarded as optional feature sets with high repeatability.

Then, variance threshold selection and correlation coefficient tests were applied to preliminarily screen the radiomics features. Subsequently, the symbolic regression method based on genetic algorithms was adopted to construct and transform the original features, thus we got ‘struct features’. Finally, recursive feature elimination with cross validation (RFECV) based on SVM algorithms was adopted to screen the remaining radiomics features to obtain a valuable feature set.

The internal five-fold validation method was used to construct the SCA differential diagnostic model as well as the MCA and IPMN differential diagnostic model. The training sets of all patients’ relevant datasets were utilized in both models, and model verification was performed on each dataset by stratified sampling. The SVM algorithm was adopted in both models. Finally, the prediction efficiency of the radiomics model was evaluated using the average AUC of the receiver operating characteristic (ROC) curve.

#### DL feature extraction

In this study, the DL features were extracted using the transfer learning (TL) method and the structure of this DL network is shown in Table S1. The public dataset [29] of breast cancer pathology sections from Kaggle were downloaded, and used for pretraining. The original data was randomly divided into training, validation and test sets at a ratio of 60%: 20%: 20% and were trained on the transformed network. After model training, DL features were extracted from the outputs of the fully connected layer. A detailed introduction of TL and the process of DL feature extraction can be found in Method S1.

#### Fused model construction and evaluation

**Fig. 1:**
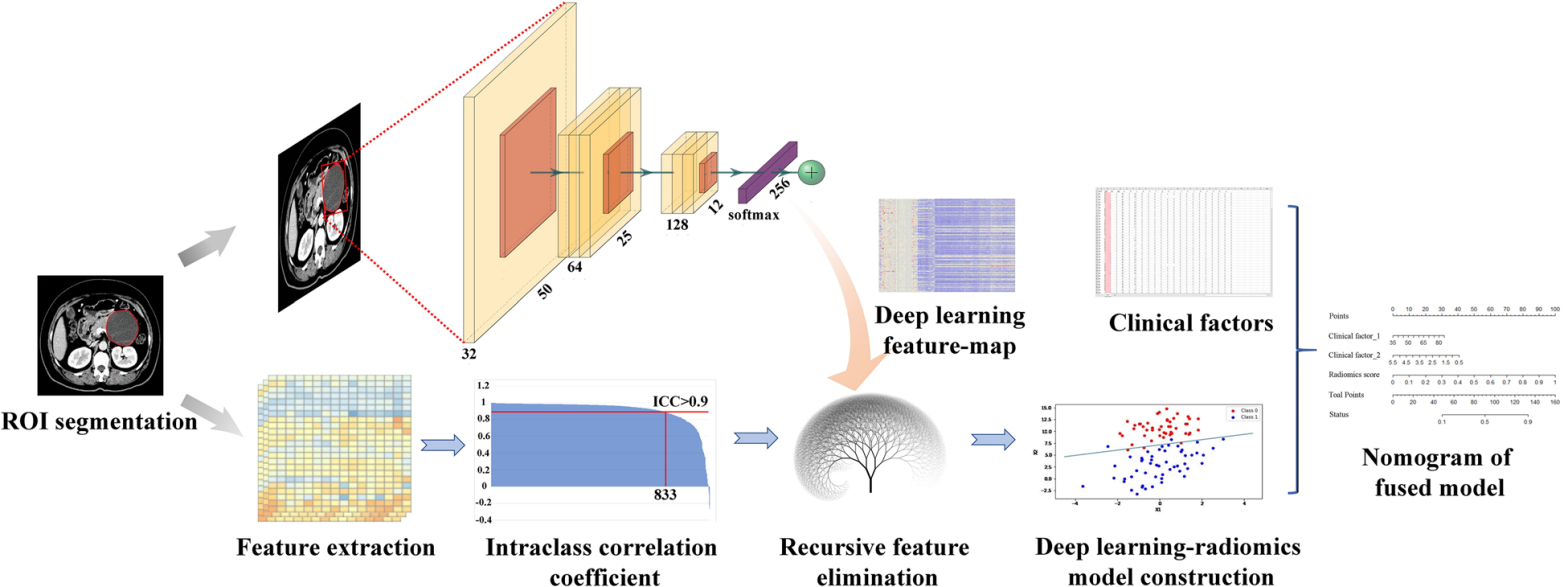
Flowchart of the deep learning radiomics research

In the SCA differential diagnostic model, the radiomics features were fused with the DL features to form the radiomics-DL feature set. In addition, the SVM classification algorithm was also used to train the radiomics-DL model using five-fold-cross-validation. The fused model was constructed by combining the clinical features, radiological features and the radiomics-DL score. As a comparison, the clinical model was also constructed by clinical characteristics and radiological features of significant difference between two groups. In terms of the MCA and IPMN differential diagnosis task, the radiomics prediction score, clinical features, and radiological features were employed to construct the fused model. The combined feature selection was completed according to AIC. In addition, the final fused model was constructed using a logistic regression method. Consistent with the radiomics model, the accuracy and AUC of the fused model were adopted for the evaluation. The flowchart of this study is shown below in Fig. 1.

### Statistical analysis

The software packages used for the data processing and statistical analysis included SPSS24 (IBM Corp, Chicago, USA), MATLAB (2018b, MathWorks, Natick, MA, USA), Python (https://www.python.org/), and R 3.6.1 (http://www.Rproject.org). The data analysis was performed with SPSS 24 (IBM Corp, Chicago, USA). The form of ‘ $\overline{\textrm{x}}\pm \textrm{s}$ ’ was used to show the comparison results of the continuous feature groups using the Mann–Whitney U test. The absolute numbers and percentages were used to show the comparison results of the features meeting discrete variable criteria using the χ^2^ and Fisher’s exact test. The criterion of statistical significance was considered p<0.05. ROCs were plotted, and AUCs were computed using SPSS24 and the R package ‘pROC’. Radiomics features were extracted with the ‘Wavelet’, ‘Communication Toolbox’ and ‘Radiomics’ standard libraries in MATLAB.

## Results

### Analysis of the clinical and imaging data

In the SCA differential diagnostic model, the clinical and radiological features of 193 patients were included in the data statistics, including 99 patients (51.8 ± 11.6 years old) with SCA and 94 patients (50.2 ± 14.3 years old) with MCA and IPMN. Among these patients, sex was the only clinical characteristic with a significant difference among the diagnostic groups (*p* < 0.05). Regarding the radiological features, position, calcification, lobulation, polycystic features (≥2, ≥6), pancreatic duct dilatation and communication with the pancreatic duct showed significant differences (p < 0.05) between the SCA and non-SCA groups, while the others did not (*p* > 0.05). The statistical analyses of the clinical and imaging features in the SCA differential diagnostic model is shown in Table S2 and Table 1, respectively.

**Table 1:** Comparison of the radiological characteristics between pancreatic serous cystadenoma (SCA) and non-SCA

Radiological Characteristics	SCA (***n =*** 99)	Non-SCA (***n =*** 94)	***p***
**Position**			0.014
Head and Neck	45 (45%)	28 (30%)	
Body and Tail	54 (55%)	66 (70%)	
**Solitary/multiple cysts**			0.001
Solitary	5 (5%)	22 (23%)	
Multiple	94 (95%)	72 (77%)	
**Maximum diameter (cm)**	4.0 ± 2.2	4.1 ± 2.4	0.871
**Lobulation**			0.001
With	69 (70%)	44 (47%)	
Without	30 (30%)	50 (53%)	
**Polycystic features (≥2)**			0.712
With	77 (78%)	71 (76%)	
Without	22 (22%)	23 (24%)	
**Polycystic features (≥6)**			0.006
With	51 (52%)	30 (32%)	
Without	48 (48%)	64 (68%)	
**Nodular soft tissue**			0.261
With	16 (16%)	10 (11%)	
Without	83 (84%)	84 (89%)	
**Cystic wall calcification**			< 0.001
With	33 (33%)	10 (11%)	
Without	66 (67%)	84 (89%)	
**Pancreatic duct dilatation**			0.001
With	5 (5%)	21 (22%)	
Without	94 (95%)	73 (78%)	
**Communication with the pancreatic duct**			0.031
With	25 (25%)	14 (15%)	
Without	69 (75%)	85 (85%)	
**Peripancreatic lymph node enlargement**			0.609
With	2 (2%)	3 (3%)	
Without	97 (98%)	91 (97%)	

In the MCA and IPMN differential diagnostic model, the clinical and radiological features of 94 patients were included in the data statistics, including 55 patients (45.9 ± 14.3 years old) with MCA and 39 patients (61.1 ± 8.3 years old) with IPMN. Sex and age were the only two characteristics with significant differences among the diagnostic groups (*p* < 0.05). Position, maximum diameter, lobulation, polycystic features (≥2, ≥6), pancreatic duct dilatation and communication with the pancreatic duct showed significant differences (p < 0.05) between MCA and IPMN. However, the other CT radiological features showed fewer differences (*p* > 0.05). The statistical results of the clinical and radiological features in the MCA and IPMN differential diagnostic models are listed in Table S3 and Table 2, respectively.

**Table 2:** Comparison of the radiological characteristics between mucinous cystadenoma (MCA) and intraductal papillary mucinous neoplasm (IPMN)

Radiological Characteristics	MCA (***n =*** 55)	IPMN (***n =*** 39)	***p***
**Position**			0.045
Head and Neck	5 (9%)	23 (59%)	
Body and Tail	50 (91%)	16 (41%)	
**Solitary/multiple cysts**			< 0.001
Solitary	4 (7%)	18 (46%)	
Multiple	51 (93%)	21 (52%)	
**Maximum diameter (cm)**	4.7 ± 2.7	3.2 ± 1.6	0.002
**Lobulation**			< 0.001
With	11 (20%)	33 (85%)	
Without	44 (80%)	6 (15%)	
**Polycystic features (≥2)**			0.014
With	36 (65%)	35 (90%)	
Without	19 (35%)	4 (10%)	
**Polycystic features (≥6)**			0.001
With	10 (18%)	20 (51%)	
Without	45 (82%)	19 (49%)	
**Nodular soft tissue**			1.000
With	6 (11%)	4 (10%)	
Without	49 (89%)	35 (90%)	
**Cystic wall calcification**			0.263
With	8 (15%)	2 (5%)	
Without	47 (85%)	37 (95%)	
**Pancreatic duct dilatation**			< 0.001
With	2 (4%)	19 (49%)	
Without	53 (96%)	20 (51%)	
**Communication with the pancreatic duct**			< 0.001
With	3 (5%)	22 (56%)	
Without	52 (95%)	17 (44%)	
**Peripancreatic lymph node enlargement**			0.568
With	1 (2%)	2 (5%)	
Without	54 (98%)	37 (95%)	

### Feature extraction and feature screening

During the extraction of the radiomics features, 1067 radiomics features were obtained from the ROIs of CT data of all 193 patients, including 7 global histogram features, 53 texture features, 159 LBP features, and 848 wavelet features. Details of the radiomics features extracted in this study are listed in Table S4. Under the condition of ICC > 0.9, 833 radiomics features passed the repeatability test.

During the construction of the DL model, the accuracy rate of the pretrained model based on breast cancer pathology data was 0.761. The accuracy rate of the SCA DL model was 0.670, and 256 DL features were collected through the final model. The heatmap of the extracted DL features is shown in Fig. S2.

**Fig. 2:**
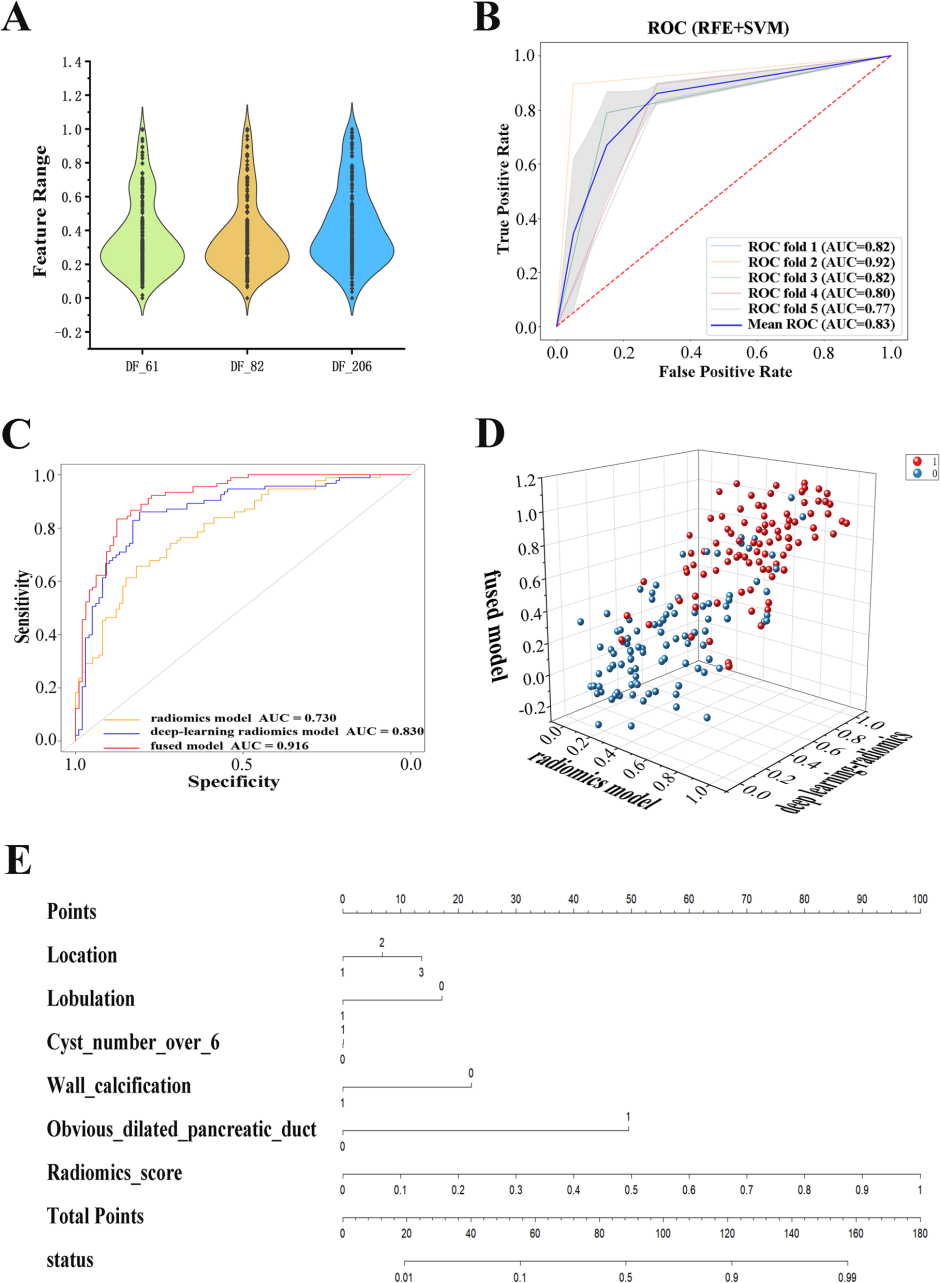
**A**: Violin plots of the feature range of the 3 most important deep learning features evaluated in construction of SCA differential diagnostic model; **B**: ROC curves of five-fold cross-validation with the deep learning radiomics model for the SCA differential diagnosis; **C**: ROC curves of the radiomics model, deep learning radiomics model and fused model for the SCA differential diagnosis; **D**: Predictions of the radiomics model, deep learning radiomics model and fused model for each patient shown as a 3D pattern; **E**: Nomogram of the fused model for the SCA differential diagnosis

Thirty struct features were synthesized based on the genetic algorithms. In the SCA differential diagnostic model, a total of 94 key features (including 2 global histogram features, 2 texture features, 57 wavelet features, 12 LBP features, 17 DL features and 4 synthetic features) were retained after RFE. Three DL features which reflect heterogeneity the most are shown in Fig. 2A. A total of 69 key features (including 2 texture features, 47 wavelet features, 6 LBP features and 14 synthetic features) were retained after RFE, and they were used to construct the MCA and IPMN radiomics diagnostic model.

### Construction and evaluation of the radiomics diagnostic models and clinical diagnostic models

**Fig. 3:**
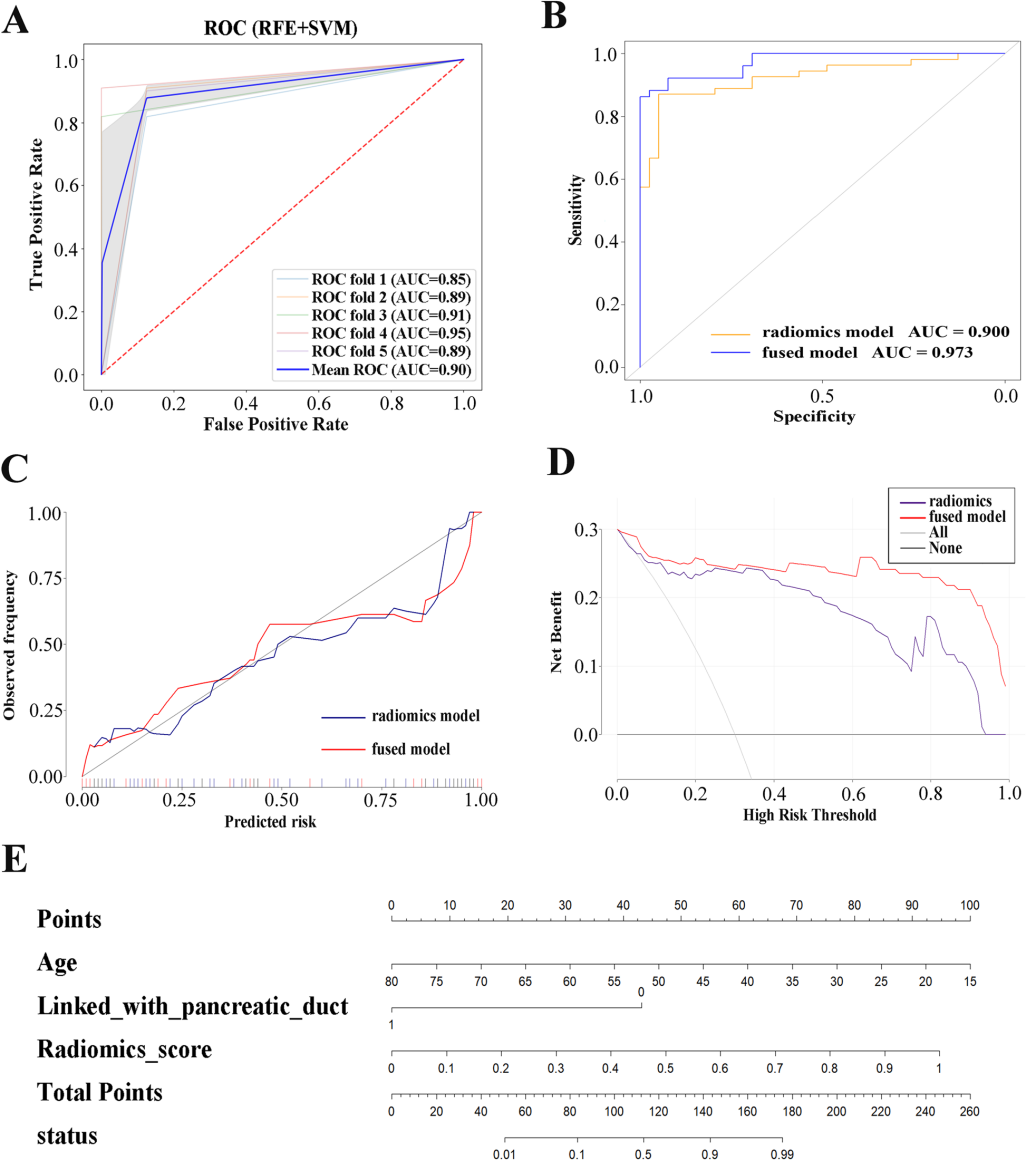
**A**: ROC curves of the five-fold cross-validation with the radiomics model for MCA and IPMN diagnosis; **B**: ROC curves of the radiomics model and fused model for MCA and IPMN diagnosis; **C**: Calibration curves of the radiomics model and fused model for MCA and IPMN diagnosis; **D**: Decision curve analysis of the radiomics model and fused model for MCA and IPMN diagnosis; **E**: Nomogram of the fused model for MCA and IPMN diagnosis

A radiomics diagnostic model with five-fold cross-validation based on SVM was established for SCA differential diagnosis. The model resulted in an average AUC of 0.730, with an accuracy of 74.0%, sensitivity of 65.6%, specificity of 81.8%, positive predictive value of 77.2% and negative predictive value of 71.7%. The average AUC of the radiomics-DL diagnostic model was 0.830, the accuracy was 83.3%, the sensitivity was 86.0%, the specificity was 80.8%, the positive predictive value was 80.8% and the negative predictive value was 86.0%. The ROC image of the five-fold cross-validation of the model is shown in Fig. 2B. A radiomics diagnostic model with five-fold cross-validation based on SVM was established for MCA and IPMN differential diagnosis. The average AUC, accuracy, sensitivity, specificity, positive predictive value and negative predictive value of the training set reached 0.900, 90.3, 87.0, 94.9, 95.9 and 84.1%, respectively. The ROC curves of the clinical models for SCA differential diagnosis as well as MCA and IPMN differential diagnosis are shown in Fig. S3. The AUCs of SCA differential diagnostic model as well as MCA and IPMN differential diagnostic model were 0.655 (95% CI: 0.536, 0.774) and 0.746 (95% CI: 0.633, 0.859), respectively. The ROC image of the fused model is shown in Fig. 3A.

### Fused model

The radiomics-DL score as well as the corresponding clinical and radiological features were included in the fused SCA differential diagnostic model. After feature selection according to the AIC, the best feature set was obtained, including the position, cyst number (≥6), wall calcification, pancreatic duct dilatation and radiomics-DL score. By means of logistic regression analyses, the AUC achieved 0.916 (95% CI: 0.876-0.955), with an accuracy of 85.6%, sensitivity of 83.3%, specificity of 87.6%, positive predictive value of 86.2% and negative predictive value of 85.0%. The AUCs of each model contributing to the SCA differential diagnosis are shown in Fig. 2C. The prediction scores of the models, including the radiomics model, radiomics-DL model and fused model were made into a scatter diagram in the same three-dimensional coordinate system as illustrated in Fig. 2D to allow for a visual evaluation of the accuracy of the model prediction. A nomogram was also constructed, as shown in Fig. 2E**,** to benefit the patient’s individualized diagnosis and treatment scheme. For model evaluation, the calibration curve and decision curve analysis of the radiomics-DL model and fused model are plotted in Fig. S4A and Fig. S4B.

In the fused MCA and IPMN differential diagnostic model, the radiomics score as well as the corresponding clinical and radiological features were included. After feature selection according to the AIC, the best feature set included age, communication with the pancreatic duct and radiomics score. After training with logistic regression, the AUC was 0.973 (95% CI: 0.947-0.999), accuracy was 92.2%, sensitivity was 86.3%, specificity was 100.0%, positive predictive value was 100.0% and negative predictive value was 84.8%. The ROC image of the fused model and radiomics model is shown in Fig. 3B. The calibration curve and decision curve analysis of the radiomics model and fused model are plotted in Fig. 3C and Fig. 3D. The nomogram is also shown in Fig. 3E for the sake of potential usage concerning clinical decision-making.

## Discussion

In this study, deep learning and radiomics methods were used to construct classification prediction models for SCA, MCA and IPMN. Then, more than 1300 radiomics features were extracted, including 256 deep learning features, which were used to construct the feature set. The fused model was constructed by logistic regression. The model established in this study achieved good results in the classification prediction of SCA, MCA, and IPMN, which proves that deep learning radiomics models have potential use in the classification of pancreatic cystic tumors.

Deep learning methods have been widely used in disease classification field. In pretraining, a transfer learning step was added to increase the dataset samples at the early stage, and some network parameters were frozen to reduce overfitting [30]. The expansion of local image data capacity can better keep the prediction efficiency of deep learning itself [31]. The fusion of deep learning features and radiomics features has been shown to be superior to deep learning or radiomics alone [32–35], which is consistent with our experimental conclusions, suggesting that there may be complementarity between deep learning features and radiomics features. We evaluated the feature importance ranking in the model by calculating and sorting the weight values of the top 30 features. We can learn from the ranking that deep learning features occupied over 50% of the 15 most valuable features. This indicates the importance of deep learning features.

The figures and results show that the radiomics-DL model is better than the radiomics model and indicate that the fused model has better predictive ability. The 3D scatter chart (Fig. 2D) shows that the predictions of the fused model are closer to the right corner of the image, which shows more stability of the prognostic results. Clinical characteristics also contain information contributing to the model construction, because the performance of the model fused with the radiomics-DL score and clinical features took first place in the AUC comparison. Evidently, comparing with the clinical-only model (AUC = 0.655), the addition of the radiomics-DL features made a huge leap for the classification and prediction of the models (AUC = 0.916) both in information supplement and interpretability. Furthermore, the valuable radiomics texture features extracted and selected showed connections with tumor heterogeneity. For instance, roughness is a valuable texture feature selected to build the SCA differentiation model, and interpretability of the radiologic features can be obtained. Roughness originally showed that the distance between the pixel gray levels can be correlated with the regional density difference of the lesions.

Our findings suggest that specific morphological features may improve the prediction efficiency for the classification of pancreatic cystic lesions. Among them, important features such as the location, number of cysts (≥6) and wall calcification were used in the fused model for SCA differential diagnosis. Unlike previous studies, in our study, tumor size was not included in the prediction model to distinguish between serous and nonserous pancreatic cystic tumors. Previous research results also confirmed that imaging features could be included in the classification prediction models of SCA and MCA and even play a role in the classification models of atypical SCA and MCA [24, 27]. Obviously, location serves as an important feature for improving the classification prediction models in radiomics. We believe the reason is that radiomic features do not include location information, and the location of the tumor as an imaging feature can improve the prediction efficiency of the models.

Communication with the pancreatic duct as an important morphological feature was included in the MCA and IPMN differential diagnostic model. The current consensus is that communication with the pancreatic duct is an important imaging feature of IPMN to distinguish it from other pancreatic cystic tumors. Yang and Shen’s previous radiomics studies did not incorporate imaging features into the feature set to construct the classification model of pancreatic cystic lesions [22, 28]. However, in our study, only communication with the pancreatic duct played a role in our MCA and IPMN differential diagnostic model. We believe this is due to the inability of radiomics features derived from IPMN tumors to reflect the tumor-pancreatic duct relationship. Therefore, in radiomics studies, the evaluation of morphological features is necessary.

As a retrospective radiomics study, this study has certain limitations. First, despite the large number of cases included in our study, this is still a single-center study and the performance of our models on other datasets is uncertain. Second, we used 2D imaging data for the convenience of clinical use, while 3D data may contain more information about tumor heterogeneity. However, our study shows that the 2D-based radiomics model also has high predictive performance for the classification of pancreatic cystic lesions. Third, we only used CT image data for model building. The classification models of pancreatic cystic lesions still need to be explored based on different imaging modalities. In the future, we will further explore the classification model of pancreatic cystic lesions based on other types of images to meet the needs of individualized clinical treatment.

## Conclusions

Our data demonstrate that a radiomics-based approach can be used for classification prediction of pancreatic cystic tumors using CT data. In addition, adding deep learning features and morphological features can further improve the prediction efficiency of the models. These two classification models will provide a noninvasive, individualized evaluation for each patient and help meet the needs of clinical precision medicine.

## Supplementary Information

**Additional file 1:  **Table S1, Table S2, Table S3, Table S4, Fig. S1, Fig. S2, Fig. S3, Fig. S4 and Method S1 is available.

## Data Availability

The data that support the findings of this study are available from the corresponding author upon reasonable requests.

## Abbreviations

PCN: Pancreatic cystic neoplasm
DL: Deep learning
CT: Computed tomography
SCA: Serous cystadenoma
MCA: Mucinous cystadenoma
IPMN: Intraductal papillary mucinous neoplasm
ROI: Region of interest
SVM: Support vector machine
AIC: Akaike information criterion
AUC: Area under the curve
MRI: Magnetic resonance imaging
SCN: Serous cystic neoplasms
MCN: Mucinous cystic neoplasms
ML: Machine learning
LBP: Local binary pattern
ICC: Intraclass correlation coefficient
RFECV: Recursive feature elimination with cross validation
ROC: Receiver operating characteristic
TL: Transfer learning
