# Supplementary material for: Classification prediction of pancreatic cystic neoplasms based on radiomics deep learning models
Source: BMC Cancer. 2022 Nov 29;22:1237. doi: 10.1186/s12885-022-10273-4 (PMC9710154; doi:10.1186/s12885-022-10273-4)
Supplement: Supplementary file 1 — Additional file 1: Table S1, Table S2, Table S3, Table S4, Fig. S1, Fig. S2, Fig. S3, Fig. S4 and Method S1 is available. [file 12885_2022_10273_MOESM1_ESM.docx]

# Supplemental material

**Tables** **2**

Table S12

Table S24

Table S35

Table S46

**Figures** **8**

Figure S18

Figure S29

Figure S310

Figure S411

**Method** **12**

Method S112

**Table S1:** Construction of convolutional neural network used in this study.

| **Layer** | **Output Shape** | **Parameters** |
| --- | --- | --- |
| separable_conv2d | 50×50×32 | 155 |
| activation (relu) | 50×50×32 | 0 |
| batch_normalization | 50×50×32 | 128 |
| max_pooling2d | 25×25×32 | 0 |
| dropout | 25×25×32 | 0 |
| separable_conv2d_1 | 25×25×64 | 2400 |
| activation_1 (relu) | 25×25×64 | 0 |
| batch_normalization_1 | 25×25×64 | 256 |
| separable_conv2d_2 | 25×25×64 | 4736 |
| activation_2 (relu) | 25×25×64 | 0 |
| batch_normalization_2 | 25×25×64 | 256 |
| separable_conv2d_3 | 25×25×64 | 4736 |
| activation_3 (relu) | 25×25×64 | 0 |
| batch_normalization_3 | 25×25×64 | 256 |
| max_pooling2d_1 | 12×12×64 | 0 |
| dropout_1 | 12×12×64 | 0 |
| separable_conv2d_4 | 12×12×128 | 8896 |
| activation_4 (relu) | 12×12×128 | 0 |
| batch_normalization_4 | 12×12×128 | 512 |
| separable_conv2d_5 | 12×12×128 | 17664 |
| activation_5 (relu) | 12×12×128 | 0 |
| batch_normalization_5 | 12×12×128 | 512 |
| separable_conv2d_6 | 12×12×128 | 17664 |
| activation_6 (relu) | 12×12×128 | 0 |
| batch_normalization_6 | 12×12×128 | 512 |
| max_pooling2d_2 | 6×6×128 | 0 |
| dropout_2 | 6×6×128 | 0 |
| flatten | 4608 | 0 |
| dense | 256 | 1179904 |
| activation_7 (relu) | 256 | 0 |
| batch_normalization_7 | 256 | 1024 |
| dropout_3 | 256 | 0 |
| dense_1 | 2 | 514 |
| activation_8 (softmax) | 2 | 0 |

**Table S2:** Comparison of the clinical characteristics between pancreatic serous cystadenoma (SCA) and non-SCA.

| **Clinical characteristics** |  | **SCA**  **(n=99)** | **Non-SCA**  **(n=94)** | ***p*** |
| --- | --- | --- | --- | --- |
| **Sex** |  |  |  | 0.024 |
|  | Male | 16 (16%) | 28 (30%) |  |
|  | Female | 83 (84%) | 66 (70%) |  |
| **Age** |  | 51.8 ± 11.6 | 50.2 ± 14.3 | 0.375 |
| **Abdominal symptoms** |  |  |  | 0.055 |
|  | With | 30 (30%) | 41 (44%) |  |
|  | Without | 69 (70%) | 53 (56%) |  |
| **History of chronic pancreatitis** |  |  |  | 0.236 |
|  | With | 0 | 2 (2%) |  |
|  | Without | 99 (100%) | 92 (98%) |  |
| **CEA*** |  |  |  | 1.000 |
|  | High | 4 (4%) | 4 (4%) |  |
|  | Normal | 93 (96%) | 87 (96%) |  |
| **CA19-9*** |  |  |  | 0.066 |
|  | High | 6 (6%) | 13 (14%) |  |
|  | Normal | 91 (94%) | 78 (86%) |  |

**Note**: CEA and CA 19-9 represent preoperative plasma carcinoembryonic antigen and carbohydrate antigen 19-9, respectively, and the reference ranges are 0-5ng/ml and 0-37U/ml, respectively. *Due to incomplete clinical data, 5 patients were not included.

**Table S3:** Comparison of the clinical characteristics between mucinous cystadenoma (MCA) and intraductal papillary mucinous neoplasm (IPMN).

| **Clinical characteristics** |  | **MCA**  **(n=55)** | **IPMN**  **(n=39)** | ***p*** |
| --- | --- | --- | --- | --- |
| **Sex** |  |  |  | <0.001 |
|  | Male | 8 (15%) | 20 (51%) |  |
|  | Female | 47 (85%) | 19 (49%) |  |
| **Age** |  | 45.9 ± 14.3 | 61.1 ± 8.3 | <0.001 |
| **Abdominal symptoms** |  |  |  | 0.396 |
|  | With | 26 (47%) | 15 (38%) |  |
|  | Without | 29 (53%) | 24 (62%) |  |
| **History of chronic pancreatitis** |  |  |  | 1.000 |
|  | With | 1 (2%) | 1 (3%) |  |
|  | Without | 54 (98%) | 38 (97%) |  |
| **CEA*** |  |  |  | 1.000 |
|  | High | 2 (4%) | 2 (5%) |  |
|  | Normal | 50 (96%) | 37 (95%) |  |
| **CA19-9*** |  |  |  | 0.517 |
|  | High | 9 (16%) | 4 (10%) |  |
|  | Normal | 43 (84%) | 35 (90%) |  |

**Note**: CEA and CA 19-9 represent preoperative plasma carcinoembryonic antigen and carbohydrate antigen 19-9, respectively, and the reference ranges are 0-5ng/ml and 0-37U/ml, respectively. *Due to incomplete clinical data, 3 patients were not included.

**Table S4:** The radiomics features used in the study.

| **Feature Category** | **Features in detail** |
| --- | --- |
| Histogram features | Mean, Variance, Deviation, Skewness, Kurtosis, Energy, Entropy, |
| Texture features | Energy, Entropy, Dissimilarity, Contrast, Inverse difference, Correlation, Homogeneity, Autocorrelation, Cluster shade, Cluster prominence, Maximum probability, Sum of squares, Sum average, Sum variance, Sum entropy, Difference variance, Difference entropy, Information measures of correlation, Information measures of correlation, Maximal correlation coefficient, Inverse difference normalized, Inverse difference moment normalized derived from GLCM.  Short Run Emphasis (SRE), Long Run Emphasis (LRE), Gray-Level Nonuniformity (GLN), Run-Length Nonuniformity (RLN), Run Percentage (RP), Low Gray-Level Run Emphasis (LGRE), High Gray-Level Run Emphasis (HGRE), Short Run Low Gray-Level Emphasis (SRLGE), Short Run High Gray-Level Emphasis (SRHGE), Long Run Low Gray-Level Emphasis (LRLGE), Long Run High Gray-Level Emphasis (LRHGE), Gray-Level Variance (GLV), Run-Length Variance (RLV) derived from GLRLM.  Small Zone Emphasis (SZE), Large Zone Emphasis (LZE), Gray-Level Nonuniformity (GLN), Zone-Size Nonuniformity (ZSN), Zone Percentage (ZP), Low Gray-Level Zone Emphasis (LGZE), High Gray-Level Zone Emphasis (HGZE), Small Zone Low Gray-Level Emphasis (SZLGE), Small Zone High Gray-Level Emphasis (SZHGE), Large Zone Low Gray-Level Emphasis (LZLGE), Large Zone High Gray-Level Emphasis (LZHGE), Gray-Level Variance (GLV), Zone-Size Variance (ZSV) derived from GLSZM.  Strength, Busyness, Complexity, Contrast, Coarseness derived from NGTDM. |
| Wavelet features | Wavelet texture features derived from images transformed in low-frequency sub-bands, horizontal high-frequency sub-bands, vertical high-frequency sub-bands and diagonal high-frequency sub-bands at successive multiscale. |
| LBP features | LBP features derived from images transformed in local binary patterns of uniform, rotation invariant and uniform rotation invariant. |

**Note:** LBP: Local Binary Pattern; GLCM: Gray-Level Co-occurrence Matrix; GLRLM: Gray-Level Run-Length Matrix; GLSZM: Gray-Level Size Zone Matrix; NGTDM: Neighborhood Gray Tone Difference Matrix.

**Figure S1**: The flowchart of population data collection process in our study.


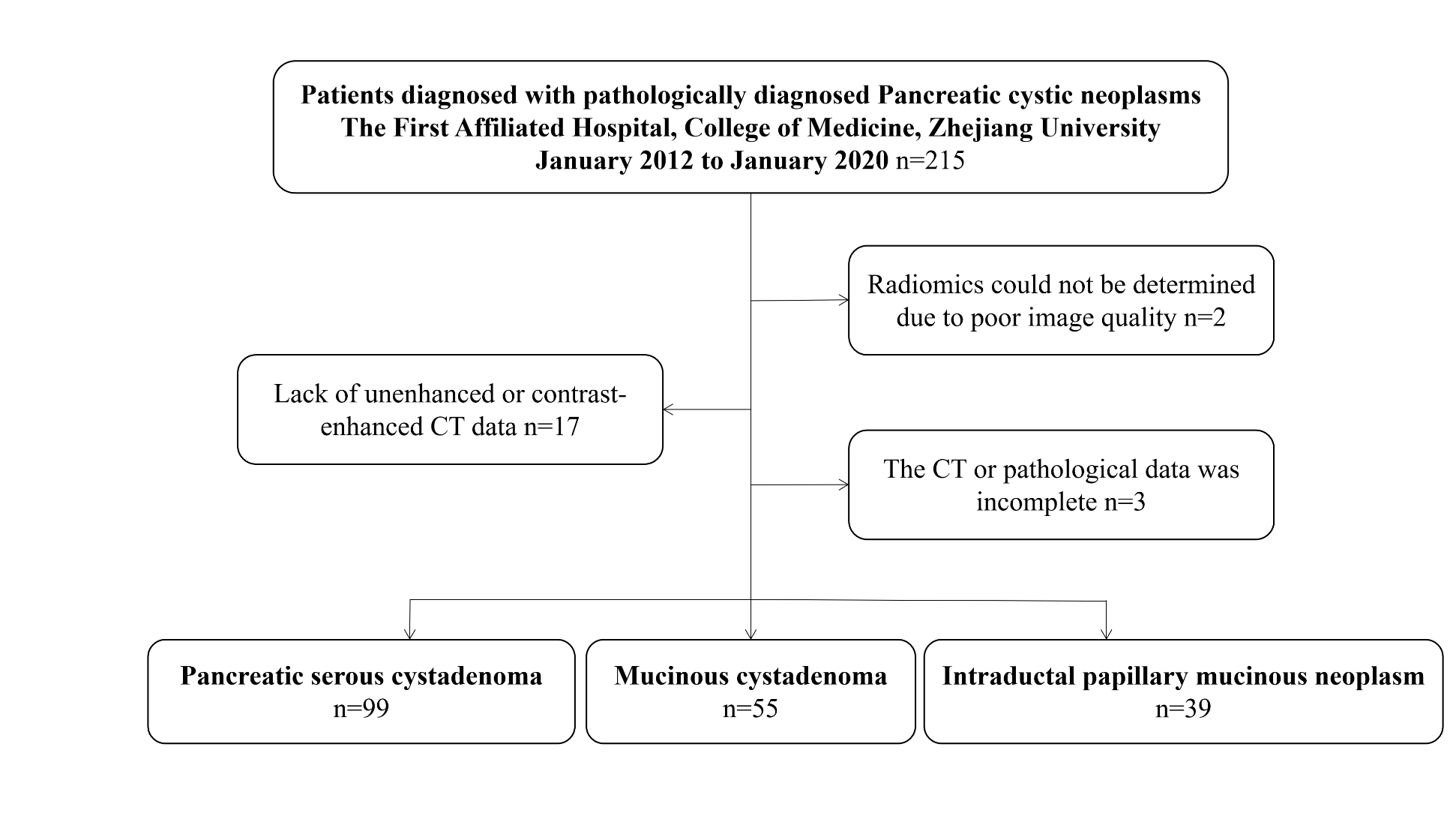


**Figure S2**: The heatmap of extracted DL features.


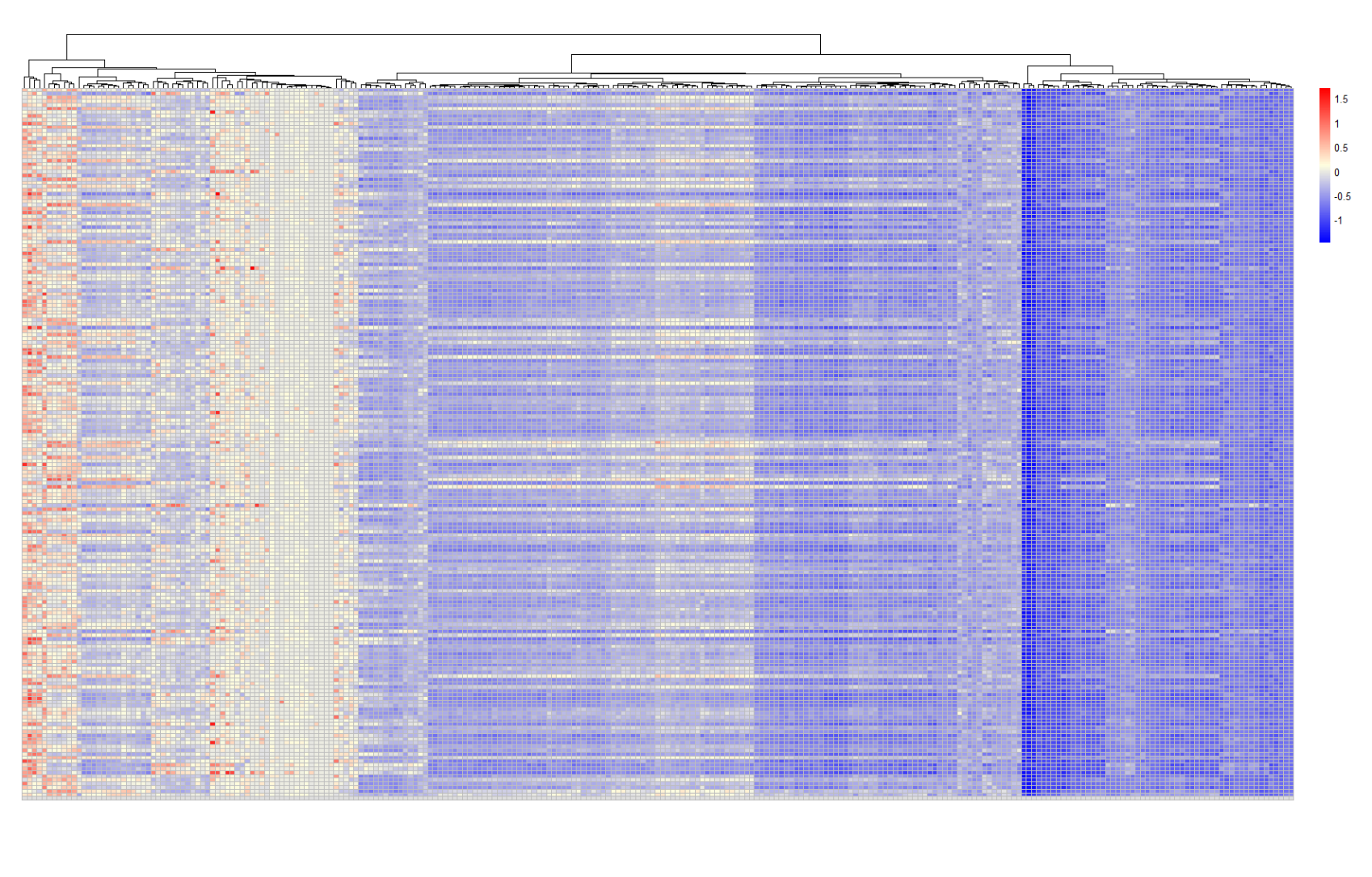


**Figure S3**: The ROC curves of the clinical models for SCA differential diagnosis as well as MCA and IPMN differential diagnosis.


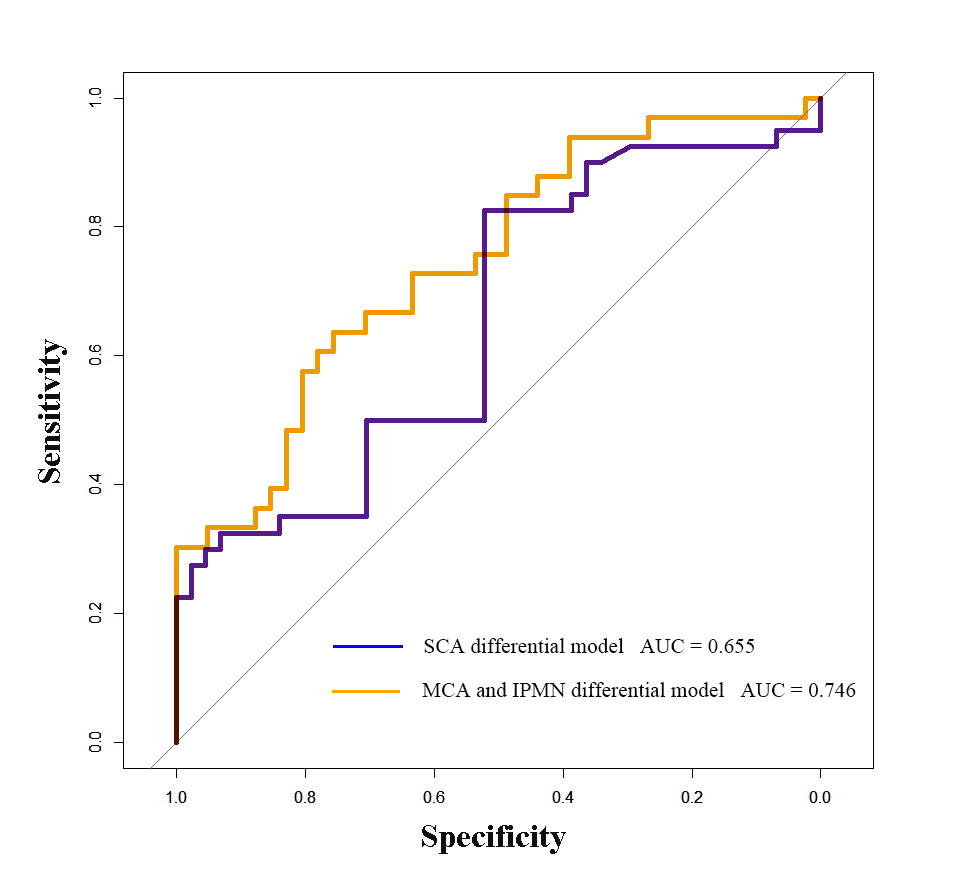


**Figure S4 A**: Calibration curve analysis of the radiomics-DL model and fused model; **B**: Decision curve analysis of the radiomics-DL model and fused model.


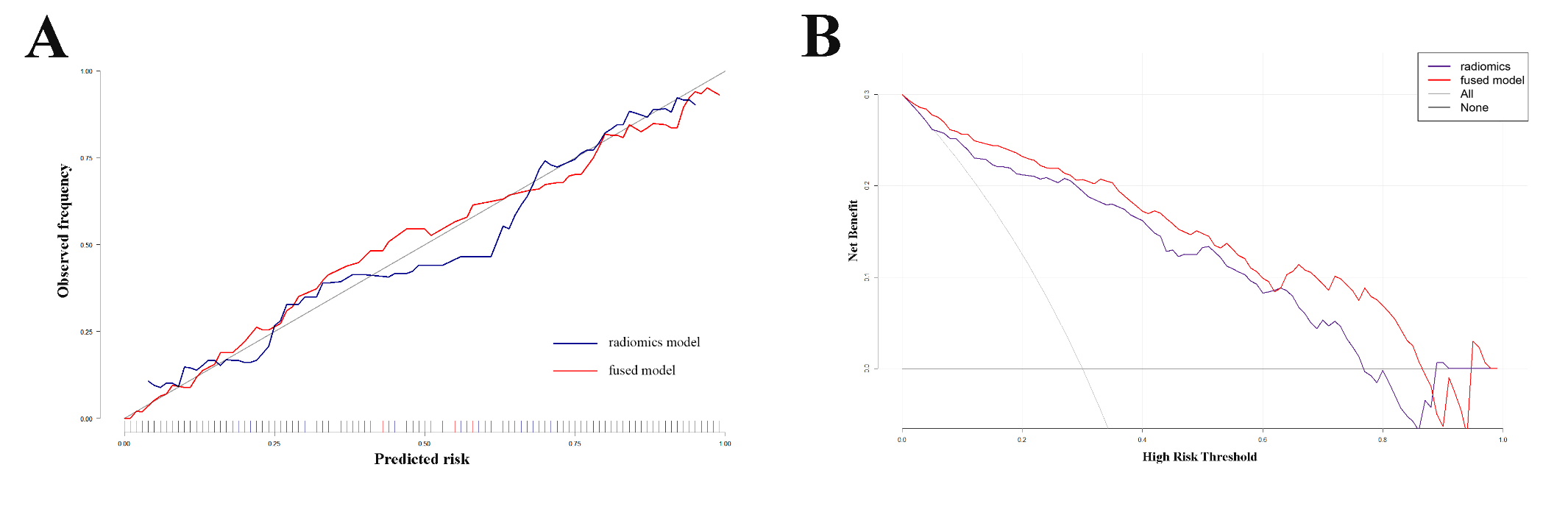


**Method S1**

In this study, DL features were extracted using transfer learning (TL) method. In deep learning studies, it is a common method to use pre-trained models as the starting point of new models in computer vision tasks. Usually, these pre-trained models have consumed plenty of time and computing resources when training neural networks. Transfer learning can transfer the parameters, namely useful knowledge to related tasks.

In our study, a DL network with a depth of 34 layers was constructed. The network included convolution, activation, batch normalization, max pooling, dropout layer, fully connected layer and the fully connected regression layer. The structure of this model is shown in **Table S1**. The ROIs segmented from the CT images of patients with PCNs were considered, and the minimum rectangular box containing a complete ROI of irregular shape was extracted. The image size was adjusted to 50×50 pixels by linear interpolation, and then three channels (R, G, B) were formed to meet the model input requirements, consistent with pre-training data. As data preprocessing steps, patches from original input images were collected for data enhancement, and 36 patch images were obtained from a single ROI by intercepting the square window with step size of 5 pixels and window width of 25 pixels. A total of 6,948 patches were obtained from all images, including 3,564 SCA patches, 1980 MCA patches and 1404 IPMN patches. Each image was resized to 50×50 pixels by linear interpolation.

The public datasets of breast cancer pathology sections from Kaggle were downloaded, and a total of 277,524 pictures were used for pre-training, including 198,738 pictures without breast cancer and 78,786 pictures with breast cancer, with a size of 50×50 pixels. Then, the training, validation and test sets were randomly split at the ratio of 60%: 20%: 20%. Subsequently, the weight value of deep learning model with the highest accuracy and without obvious overfitting was selected and retained. The weight parameters of the first 11 layers were fixed in this model, and the same model structure with fixed parameters was used to perform the training of local datasets. All patients were divided into the training (60%), validation (20%), and test sets (20%). Then, the patch images corresponding to these patients were randomly divided into the training, validation and test sets. The model training results were obtained after TL, and outputs of the 29th layer (the fully connected layer) were fixed and extracted as DL features conforming to the model. Hence, DL features were obtained.
